# From Readiness Assessment to Co-Design: A Cross-Sectional, Living Lab-Informed Study of Telemedicine Implementation in Albania

**DOI:** 10.3390/nursrep16100350

**Published:** 2026-09-26

**Authors:** Blerina Duka, Michele Biring-Pani, Fabiola Mane, Jola Çini, Armela Zylfo, Kujtime Vakeflliu, Ippolito Notarnicola

**Affiliations:** 1 Department of Medicine, Barleti University, 1000 Tirana, Albania; m.biring@umb.edu.al (M.B.-P.); koord.mjekesi-tek@umb.edu.al (F.M.); j.xharo@umb.edu.al (J.Ç.); a.zylfo@umb.edu.al (A.Z.);; 2 Department of Medicine and Surgery, University of Enna “Kore”, 94100 Enna, Italy

**Keywords:** telemedicine, living lab, digital health, healthcare professionals, implementation readiness, professional acceptance

## Abstract

**Background/Objectives:** Telemedicine has substantial potential to improve access and continuity of care, but implementation depends on professional, organizational, technological, and governance readiness. This cross-sectional study examined healthcare professionals’ perceptions of telemedicine implementation in Albania as an exploratory needs-assessment phase informed by Living Lab principles. **Methods:** An online survey was completed by 1213 healthcare professionals. The study used a questionnaire developed for this project; its multi-item domains are therefore treated as exploratory rather than established constructs. Descriptive statistics, exploratory factor analysis, Spearman correlations, and multivariable linear and logistic regression were used. Two supplementary open-ended questions were analyzed using content-oriented thematic coding and integrated with the structured survey findings to identify preliminary priorities for later participatory co-design. **Results:** High future willingness to use telemedicine was reported by 67.4% of participants. Continuous training (84.7%), adaptation of traditional healthcare delivery (65.8%), limited digital skills (64.7%), and lack of clear protocols/guidelines (61.5%) were frequently endorsed implementation issues. Perceived usefulness showed the strongest adjusted statistical association with future willingness in the linear model (B = 0.665, 95% CI 0.602–0.727, *p* < 0.001) and logistic sensitivity model (OR = 5.41, 95% CI 4.32–6.79, *p* < 0.001). Professional confidence was positively associated with willingness, whereas workflow and relational barriers were negatively associated. **Conclusions:** The findings describe the perceptions of this non-probability sample and should not be interpreted as national estimates of telemedicine readiness. They provide exploratory evidence that may inform subsequent stakeholder consultation, instrument validation, and participatory Living Lab activities focused on training, workflow integration, infrastructure, governance, and patient engagement.

## 1. Introduction

Digital health is increasingly recognized as a strategic priority for strengthening health systems, improving access to care, supporting continuity of services, and responding to pressures related to workforce shortages, population aging, chronic disease burden, and geographical inequities [1]. Within this broader transformation, telemedicine is one of the most visible forms of digital health innovation, enabling professionals and patients to interact, monitor health conditions, exchange clinical information, and support decision-making across distance [2,3]. The COVID-19 pandemic accelerated telemedicine adoption globally, but also exposed a fundamental implementation gap: technological potential does not automatically translate into implementation readiness. Long-term integration depends on organizational capacity, professional acceptance, regulatory clarity, infrastructure, workflow compatibility, and sustained stakeholder engagement [4,5].

International policy documents increasingly emphasize that telemedicine should not be understood merely as a technical solution, but as a complex health service innovation requiring governance, planning, evaluation, and system-level integration [6]. The World Health Organization has highlighted the importance of digital health strategies that strengthen health systems rather than simply digitizing existing practices [7]. Similarly, recent WHO/Europe guidance on telemedicine stresses the need for assessment of the healthcare ecosystem, strategic visioning, organizational change management, service development, and continuous monitoring and optimization [8]. This perspective is particularly relevant for countries and health systems in transition, where telemedicine may improve access to specialized services and reduce geographical barriers, but where implementation may be constrained by fragmented infrastructure, limited digital competence, uncertain reimbursement mechanisms, and incomplete regulatory frameworks [9].

Albania provides a relevant context for examining these issues. Previous initiatives, including the Integrated Telemedicine and e-Health Program of Albania, have demonstrated the potential of telemedicine to improve access to specialized care, particularly for remote and regional hospitals [10,11]. However, the existence of telemedicine infrastructure or pilot programs does not automatically guarantee sustainable adoption [12]. As in other health systems, the implementation of telemedicine in Albania requires attention to the everyday realities of healthcare professionals, including their perceived needs, expectations, barriers, digital confidence, and readiness to integrate remote care into clinical workflows [10]. For this reason, a purely top-down implementation model may be insufficient. Telemedicine development requires a participatory approach able to transform professionals from passive end-users into active co-designers of digital health transformation [13].

Earlier Albanian telemedicine research has primarily examined service utilization and clinical or organizational outcomes rather than broad workforce perceptions of implementation readiness. Latifi et al. [10] analyzed teleconsultation activity from Albania and Cabo Verde using data collected between 2014 and 2018, focusing on consultation volumes, local telemedicine champions, and health-system needs. In Albania specifically, Latifi et al. [11] examined 2724 patient teleconsultations from regional and remote hospitals between 2014 and 2018 to assess access to specialist care and avoidance of unnecessary transfer, while Olldashi et al. [14] focused on 590 neurotrauma teleconsultations and transfer decisions within the nationwide teleneurotrauma network. More recently, Bode et al. [15] considered telemedicine in relation to regional healthcare efficiency and service optimization in Albania. These studies provide important local evidence that telemedicine barriers, service needs, and implementation challenges were already present before the current survey. The contribution of the present 2026 study is therefore not to claim the first evidence of telehealth barriers in Albania, but to provide broader exploratory mapping across a large, multi-professional sample and multiple care levels, covering professional readiness, perceived usefulness, workflow and relational issues, technological and governance constraints, future willingness, and participant-generated priorities for later co-design.

The Living Lab approach offers a promising methodological and conceptual framework for this purpose [16]. Living Labs are commonly described as user-centered, open innovation ecosystems in which stakeholders collaborate to co-create, test, and refine innovations in real-life contexts [17,18]. In healthcare, Living Labs can support the development of digital health solutions by bringing together professionals, patients, researchers, managers, technology developers, and policy actors in iterative processes of exploration, co-design, experimentation, evaluation, and scaling [19]. Rather than isolating innovation from practice, Living Labs embed innovation within the settings where care is actually delivered. This is particularly important for telemedicine, because its success depends not only on software or devices, but also on trust, professional identity, clinical routines, patient–professional relationships, data governance, legal accountability, and institutional support.

Recent literature highlights a second polarity: technological innovation versus organizational and relational transformation. Healthcare professionals frequently identify infrastructure limitations, technical problems, workload, insufficient training, legal uncertainty, privacy concerns, and limited managerial support as barriers [20,21]. Conversely, training opportunities, perceived usefulness, organizational support, stakeholder involvement, and alignment with clinical needs may facilitate adoption [22]. Telemedicine readiness should therefore be viewed as an exploratory, multidimensional implementation condition shaped by professional capability, organizational culture, technological resources, governance, and care workflows rather than as a single individual attitude [23].

A third polarity concerns readiness assessment versus participatory co-design. Readiness assessment can identify perceived barriers and priorities, but it is not equivalent to co-design. Genuine co-design requires iterative interaction among stakeholders, shared problem definition, development of solutions, testing, feedback, and refinement [24,25]. A Living Lab perspective is useful precisely because it can connect an initial needs-assessment phase with later participatory stages in real-world settings [26]. Accordingly, the present survey should be understood as an exploratory precursor to co-design, not as evidence that a complete Living Lab or co-design intervention has already been undertaken.

In this study, telemedicine implementation in Albania is examined through a Living Lab-informed lens. Healthcare professionals are treated as key informants whose perceptions may guide later participatory development. The survey represents an exploratory needs-assessment phase aimed at describing perceived barriers, readiness indicators, and potential priorities for subsequent co-design. This framing aligns reader expectations with the cross-sectional nature of the data while retaining the Living Lab as the prospective implementation pathway.

Therefore, the aim of this cross-sectional study was to explore healthcare professionals’ perceptions of telemedicine implementation in Albania and to identify preliminary priorities that may inform a subsequent Living Lab process. Specifically, the study sought to describe perceived technological, educational, organizational, relational, legal, and policy-related implementation issues; examine factors statistically associated with future willingness to use telemedicine; and integrate structured survey findings with open-ended responses to generate stakeholder-informed priorities for future participatory work. The study does not estimate national telemedicine readiness and does not claim that co-design itself was conducted.

## 2. Materials and Methods

### 2.1. Study Design

This study adopted a cross-sectional survey design incorporating structured quantitative items and two supplementary open-ended questions. The Living Lab framework informed the interpretation and future orientation of the study, but the empirical component was a readiness/needs-assessment survey rather than a completed Living Lab intervention. The overall workflow comprised recruitment and online data collection, exploratory analysis of the questionnaire structure, multivariable association analyses, content-oriented coding of open-ended responses, and integration of both strands to identify preliminary priorities for later participatory co-design.

The study was reported in accordance with the Strengthening the Reporting of Observational Studies in Epidemiology (STROBE) guidelines for cross-sectional studies [27].

The Living Lab perspective guided the study in three ways. First, the questionnaire focused on real-world implementation barriers and facilitators perceived by professionals working in different care settings. Second, the structured items covered technological, educational, organizational, relational, legal, policy, and professional-readiness issues. Third, the two open-ended questions captured suggestions for future interventions. These elements were integrated only at the interpretation stage and were used to inform, rather than constitute, subsequent co-design activities (Figure 1).

### 2.2. Setting

The study was conducted in Albania and involved healthcare professionals working across different regions and levels of care. The Albanian healthcare system represents a relevant context for exploring telemedicine implementation because digital health solutions may contribute to improving access to care, continuity of services, and support for underserved or geographically remote areas. However, the successful integration of telemedicine requires a careful understanding of local technological, professional, organizational, and regulatory conditions.

Data were collected through an online questionnaire between January and February 2026, according to the timestamps recorded in the survey dataset. The questionnaire was administered in Albanian.

### 2.3. Participants and Sampling

The target population consisted of healthcare professionals working in the Albanian healthcare system. Eligible participants included nurses, physicians, midwives, laboratory technicians, imaging technicians, physiotherapists, speech therapists, and other healthcare professionals involved in clinical or healthcare service delivery. Participants were eligible if they were currently practicing as healthcare professionals and were able to complete the questionnaire in Albanian.

A non-probability sampling strategy was used. Participants were invited through institutional communication channels and professional networks that distributed the online survey link to eligible healthcare professionals. Because invitations could be redistributed within professional networks, the number of individuals exposed to the invitation was not known and a response rate could not be calculated. The available study records did not support a reliable reconstruction of the number or timing of follow-up reminders; therefore, no reminder frequency is reported.

Recruitment was therefore open to eligible healthcare professionals reached through the participating institutional and professional channels rather than to a pre-enumerated sampling frame. Eligibility required current practice as a healthcare professional in Albania and ability to complete the Albanian-language questionnaire. No quota sampling or profession-specific enrollment target was applied.

Participation was voluntary. No exclusion criteria were applied based on age, gender, profession, geographical area, level of care, or previous experience with telemedicine, provided that the participant belonged to the healthcare workforce.

A total of 1213 responses were collected and included in the dataset. Before analysis, data were screened for completeness, plausibility, and consistency. Implausible values, particularly for age, were checked and managed according to predefined data-cleaning criteria.

Participant-flow and data-quality checks were further examined for this revision. The analytical dataset contained 1213 submitted records. Because the survey was anonymous and no direct personal identifiers were retained in the analytical dataset, deterministic adjudication of repeat participation was not possible. Post hoc screening identified four pairs of records with identical response profiles across all survey variables except timestamp; the paired submissions occurred 3 to 137 s apart. These records were retained in the primary analyses because identical de-identified response profiles could not be conclusively classified as repeat submissions. As a sensitivity check, exclusion of the later record in each pair yielded *n* = 1209 and changed the key descriptive agreement percentages by no more than 0.14 percentage points, indicating no material effect on the descriptive conclusions. Missingness was minimal for the variables used in multivariable modeling: one participant had missing data for years of professional experience, resulting in complete-case *n* = 1212 for the regression models; no imputation was performed. Region of work was available for 770 participants and was therefore not included in the final multivariable models. Each open-ended prompt contained 1206 substantive responses (99.4% of the full sample); seven blank or non-substantive responses per prompt were excluded from content coding.

### 2.4. Questionnaire Development and Structure

Data were collected using a structured questionnaire developed specifically for this study to assess healthcare professionals’ perceptions of telemedicine implementation within a Living Lab-informed framework.

Questionnaire development followed a conceptually driven process. An initial review of the literature was conducted focusing on telemedicine implementation, digital health readiness, technology acceptance, organizational readiness, professional acceptance, and Living Lab approaches in healthcare. Relevant international guidance and previous empirical studies were also considered to identify recurring determinants of telemedicine implementation. On this basis, an initial pool of items was generated to cover the principal technological, educational, organizational, relational, governance, legal, policy, and professional-readiness dimensions considered relevant to the Albanian healthcare context.

The draft questionnaire was subsequently reviewed by healthcare and academic experts with experience in digital health, telemedicine, healthcare organization, and health services research. The expert review focused on the relevance of each item to the intended conceptual domain, clarity of wording, comprehensibility, redundancy, and contextual appropriateness for healthcare professionals working in Albania. Based on this review, minor revisions were made to improve wording, reduce ambiguity, and enhance the overall comprehensibility of the questionnaire before administration. The final version was administered in Albanian.

The available study documentation does not contain a separate formal pilot-testing sample or a standalone pilot psychometric dataset. Accordingly, no pilot reliability or validity estimates are reported. The instrument-development sequence is therefore described as literature-informed item generation, expert review for relevance and clarity, refinement of wording and redundancy, and finalization of the Albanian online form before field administration. Empirical dimensionality and internal consistency were evaluated only after full-sample data collection and are explicitly treated as exploratory. For transparency and reproducibility, the complete study questionnaire is provided as Supplementary Material File S1, including the original Albanian wording and an English translation of all sociodemographic, professional, Likert-scale, and open-ended items.

The questionnaire comprised three main sections. The first section collected sociodemographic and professional characteristics, including profession, region of work, age, educational level, years of professional experience, level of care, and previous experience with telemedicine. Profession was classified into physician, nurse, midwife, laboratory technician, imaging technician, physiotherapist, speech therapist, and other healthcare professions. Professional experience was categorized as <5 years, 5–10 years, 11–20 years, and >20 years, while level of care was classified as primary, secondary, tertiary, or other.

Previous experience with telemedicine was assessed using a dichotomous item indicating whether participants had previously used or worked with telemedicine. This variable was retained because prior exposure may influence perceived usefulness, professional confidence, and willingness to use telemedicine in future practice.

The second section included Likert-scale items assessing perceived barriers, facilitators, and professional readiness related to telemedicine implementation. Responses were recorded on a five-point agreement scale ranging from “Aspak dakord” to “Plotësisht dakord”, corresponding to “strongly disagree” and “strongly agree”. Responses were coded from 1 to 5, with higher scores indicating stronger agreement with each statement.

Items were initially organized into theoretically defined domains reflecting the conceptual framework adopted for the study: (1) technological and infrastructural barriers; (2) training and digital competence; (3) organizational and workflow-related barriers; (4) relational and service-related barriers; (5) governance, legal, and policy barriers; and (6) perceived usefulness and professional readiness. One additional item assessed professional confidence in using telemedicine in clinical practice and was treated separately because it represented self-perceived capability rather than an implementation barrier.

The third section consisted of two open-ended questions. Participants were asked to identify the main barriers to integrating telemedicine within their institution and to indicate which measures or interventions they considered most useful for effective telemedicine implementation. These questions were included to complement the structured items and to generate stakeholder-derived priorities for subsequent Living Lab co-design activities.

Because the questionnaire was developed specifically for this study, the resulting item groupings and composite domains are considered exploratory measurement structures rather than established latent constructs. The present analyses provide initial internal-structure evidence only; confirmatory factor analysis in an independent sample, convergent/discriminant validity, test–retest reliability, and measurement invariance remain necessary before the domains can be treated as validated constructs.

### 2.5. Living Lab-Informed Analytical Framework

The Living Lab framework was used to interpret the questionnaire as part of an early-stage participatory innovation process. Specifically, the study corresponded to the exploratory phase of the Living Lab cycle, during which stakeholders’ perceptions are collected to identify contextual needs, implementation barriers, and potential areas for co-design.

In this study, the Living Lab logic was operationalized through four analytical dimensions. The first dimension was professional readiness, referring to healthcare professionals’ willingness, confidence, previous experience, and perceived usefulness of telemedicine. The second dimension was implementation barriers, including technological, educational, organizational, relational, legal, and policy-related constraints. The third dimension was co-design priorities, derived from open-ended responses regarding measures considered necessary for effective implementation. The fourth dimension was system-level adaptation, referring to how telemedicine implementation may require changes in workflows, governance, infrastructure, training, and professional roles.

This framework allowed the analysis to connect individual perceptions with broader system-level implementation conditions.

### 2.6. Quantitative Data Analysis

Quantitative data were analyzed using IBM SPSS Statistics for Windows, version 26.0 (IBM Corp., Armonk, NY, USA). Descriptive statistics were used to summarize participants’ sociodemographic and professional characteristics and responses to questionnaire items. Categorical variables were reported as frequencies and percentages, whereas continuous variables were summarized using means and standard deviations or medians and interquartile ranges, as appropriate.

Likert-scale items were coded from 1 to 5, with higher scores indicating stronger agreement with each statement. Because the questionnaire was developed specifically for the present study, its dimensional structure was empirically examined before the domain-based composite scores were used in subsequent multivariable analyses.

The dimensionality assessment focused on the 23 Likert-scale items contributing to the multi-item implementation groupings. The single items assessing professional confidence and future willingness were excluded from factor analysis because they were treated separately in subsequent association models. No confirmatory factor analysis is presented in this study; accordingly, the factor solution is interpreted as exploratory and hypothesis-generating.

Sampling adequacy for factor analysis was assessed using the Kaiser–Meyer–Olkin (KMO) measure, and the factorability of the correlation matrix was evaluated using Bartlett’s test of sphericity. The number of factors to retain was informed by parallel analysis, together with examination of the scree plot and theoretical interpretability of the resulting solution.

Exploratory factor analysis (EFA) was performed using an oblique rotation because implementation dimensions were expected to correlate. Factor loadings, communalities, cross-loadings, and conceptual coherence were considered jointly. Statistical co-loading was not treated as sufficient evidence that conceptually distinct statements represented a single validated barrier construct. In particular, items concerning training need, digital-skill deficits, and adaptation of traditional care delivery were retained as an exploratory empirical grouping but are interpreted primarily at item level in the substantive results and discussion.

Internal consistency was assessed for the exploratory multi-item groupings using Cronbach’s alpha. Alpha coefficients are reported as descriptive reliability indicators and are not interpreted as evidence of construct validity. Composite scores were retained for exploratory association analyses, while interpretation of conceptually heterogeneous groupings was cross-checked against the constituent items.

For association analyses, the provisional multi-item groupings were operationalized as arithmetic mean composite scores calculated from the retained 1–5 Likert items, thereby preserving the original response metric. Items excluded from a composite because of insufficiently specific factorial association were not included in that score. No regression-based or Bartlett factor-score estimates were used. The resulting scores should therefore be interpreted as exploratory summary indices derived from the EFA-informed item groupings, not as confirmed latent-variable scores.

Associations between the exploratory questionnaire groupings and future willingness were initially examined using Spearman’s rank correlations. Profession, level of care, years of professional experience, educational level, and previous telemedicine experience were retained as contextual covariates because they were specified a priori as potentially relevant to telemedicine exposure, work context, or implementation perceptions. Region and age were not included in the final multivariable models because regional data were available for only 770 participants and age contained a substantial number of non-standard or implausible entries requiring data cleaning. The final models were therefore restricted to variables with sufficiently complete and interpretable data.

The primary outcome was future willingness to use telemedicine, measured on a five-point Likert scale. The multivariable linear model was used as the primary association analysis because it retained the information in the five response categories. Predictors were selected a priori from the study framework and comprised previous telemedicine experience, perceived usefulness, professional confidence, exploratory implementation groupings, profession, level of care, professional experience, and educational level. The models were intended to estimate cross-sectional associations, not causal effects or temporal predictors.

Model specification was prespecified from the study framework rather than selected to confirm the factor solution. The linear model was the primary exploratory association model because it retained the five ordered willingness categories, while the dichotomized logistic model was used only as a sensitivity analysis. All regression coefficients are therefore interpreted as cross-sectional associations among observed questionnaire measures and covariates. They are not presented as confirmation of construct validity, causal effects, or prospective prediction of telemedicine adoption.

As a sensitivity analysis, willingness was dichotomized into high willingness (“agree” or “strongly agree”) versus all other responses, and a multivariable logistic regression model was fitted using the same conceptual set of predictors. Odds ratios (ORs) and 95% confidence intervals (CIs) were reported. Following refinement of the questionnaire domains based on the dimensionality analysis, the multivariable models were re-estimated using the revised composite scores to assess whether the principal findings were robust to the empirically supported domain structure.

Prior to interpretation of the multivariable linear regression model, multicollinearity was assessed using variance inflation factors (VIFs), influence was examined using Cook’s distance, and heteroscedasticity was evaluated through residual inspection and the Breusch–Pagan test. Because heteroscedasticity was detected, HC3 heteroscedasticity-consistent robust standard errors were used. Complete-case analysis was applied to model variables. Both the linear and logistic models included *n* = 1212 participants; therefore, one of the 1213 survey records (0.08%) was excluded because of missing data in a model variable. Reference categories were nurse, primary care, <5 years of professional experience, and Bachelor degree.

All statistical tests were two-sided, and statistical significance was set at *p* < 0.05.

### 2.7. Analysis of Open-Ended Responses

Responses to the two open-ended questions were analyzed using content-oriented thematic coding rather than formal standalone thematic analysis. The purpose was descriptive: to identify recurrent implementation issues and suggested actions that could contextualize the structured survey findings and inform later stakeholder discussions.

Responses were read for familiarization, meaningful units were identified, and recurrent content was organized into a structured category framework covering infrastructure, training/digital competence, legal-regulatory clarity, protocols/guidelines, institutional support, financial/reimbursement issues, data security/privacy, resistance or acceptance of change, and patient access/awareness. Coding was performed by two coders using NVivo 15.6.0 (Lumivero, Denver, CO, USA). Categories were not mutually exclusive, so a single response could receive more than one code. The frequencies reported below therefore represent coded mentions rather than mutually exclusive groups of respondents. Blank or non-substantive text was not assigned a category. Because this component was designed as supplementary content mapping rather than an independent qualitative study, no formal intercoder-reliability coefficient was calculated.

Both open-ended prompts were optional. Substantive responses were provided by 1206 of 1213 participants (99.4%) for the question on implementation barriers and by 1206 of 1213 participants (99.4%) for the question on suggested measures/interventions. The two coders used the shared category framework in NVivo and reviewed category application iteratively. Differences in category assignment identified during review were discussed and resolved by consensus before final frequencies were produced; category definitions were clarified when necessary and affected responses were revisited. Because the purpose was descriptive content mapping rather than formal qualitative inference, no kappa or other intercoder-reliability coefficient was calculated.

The content-oriented findings were used only to complement and contextualize the quantitative results. They were not treated as an independent qualitative dataset capable of supporting theory generation. Their role was to identify recurrent participant-generated priorities for possible future Living Lab activities.

### 2.8. Integration of Structured and Open-Ended Survey Findings

Structured survey and open-ended findings were analyzed separately and integrated during interpretation. The structured items quantified the frequency and intensity of perceived implementation issues and readiness indicators; the open-ended responses provided descriptive context and participant-generated suggestions. This integration was used to identify preliminary priorities for future consultation rather than to claim completion of a mixed-methods or co-design process.

Integration was conducted at the domain level by comparing the direction and prominence of structured survey findings with the recurrent categories from the two open-ended prompts. A qualitative category was used to contextualize a quantitative finding when both addressed the same implementation issue (for example, continuous training and digital competence; legal/regulatory clarity; protocols; institutional support; or infrastructure). The open-ended material was not used to validate the quantitative constructs or to create new statistical variables; rather, it provided participant-generated context for interpreting which implementation issues might merit later stakeholder discussion and co-design.

Integration was conducted by mapping quantitative domains and qualitative themes onto the Living Lab analytical framework. For example, high agreement with the need for continuous training was interpreted together with open-ended responses emphasizing staff education and digital competence. Similarly, quantitative findings related to legal uncertainty, protocols, and institutional support were interpreted together with qualitative themes concerning regulation, policy, and managerial commitment.

This integration allowed the study to translate readiness-assessment findings into preliminary priorities that may inform subsequent participatory co-design activities within a future Living Lab process.

### 2.9. Ethical Considerations

The study was conducted in accordance with the Declaration of Helsinki and received formal ethical approval from the Ethics and Equal Opportunities Council (KESB) of Barleti University, Tirana, Albania, for the project “Assessment of Healthcare Professionals’ Perceptions and Challenges Regarding the Use of Telemedicine” (approval No. 1650/1; 12 December 2025). The Council reviewed the study protocol, accompanying documentation, and questionnaire. Participation was voluntary and anonymous. Before accessing the survey, participants received information on the study purpose, voluntary participation, confidentiality, data use, and data-protection procedures. No personally identifiable information was collected. Completion and submission of the online questionnaire constituted informed consent.

## 3. Results

For clarity, results are presented in the following sequence: participant characteristics; exploratory questionnaire structure; item-level implementation perceptions; willingness and perceived usefulness; multivariable association analyses; open-ended-response mapping; and final integration within the Living Lab-informed framework.

### 3.1. Participants’ Characteristics

A total of 1213 healthcare professionals completed the questionnaire and were included in the analysis. The sample was predominantly composed of nurses (*n* = 892; 73.5%), followed by imaging technicians (*n* = 75; 6.2%), physicians (*n* = 68; 5.6%), laboratory technicians (*n* = 62; 5.1%), midwives (*n* = 60; 4.9%), physiotherapists (*n* = 27; 2.2%), other healthcare professionals (*n* = 22; 1.8%), and speech therapists (*n* = 7; 0.6%). Most participants worked in primary healthcare settings (*n* = 758; 62.5%), followed by secondary care (*n* = 256; 21.1%), other settings (*n* = 113; 9.3%), and tertiary care (*n* = 86; 7.1%).

Regarding educational level, 526 participants (43.4%) held a bachelor’s degree, 335 (27.6%) had a professional master’s degree, 328 (27.0%) had a Master of Science degree, and 24 (2.0%) reported a doctoral degree. Professional experience was broadly distributed: 326 participants (26.9%) had less than 5 years of experience, 255 (21.0%) had 5–10 years, 313 (25.8%) had 11–20 years, and 318 (26.2%) had more than 20 years of experience. Previous experience with telemedicine was reported by 449 participants (37.0%), whereas 764 participants (63.0%) had no prior experience with telemedicine.

Regional information was available for 770 participants. Among those who reported their region of work, the most frequently represented regions were Durrës, Elbasan, Lezhë, Tiranë, Shkodër, Korçë, and Gjirokastër. Age data were screened for plausibility because some respondents entered year of birth or non-standard values rather than age. After excluding implausible entries, the valid age range indicated a predominantly adult and professionally active sample (Table 1).

### 3.2. Dimensional Structure and Internal Consistency of the Questionnaire

Because the questionnaire was developed specifically for this study, the dimensional analysis is reported as exploratory. The 23 implementation items were examined to identify provisional empirical groupings for descriptive and exploratory association analyses; professional confidence and future willingness were treated as separate single-item variables.

The data demonstrated very good suitability for factor analysis. The Kaiser–Meyer–Olkin measure of sampling adequacy was 0.905, and Bartlett’s test of sphericity was statistically significant (χ^2^(253) = 11,061.66, *p* < 0.001), indicating that the correlation matrix was appropriate for factor extraction. Parallel analysis supported the retention of five factors.

Exploratory factor analysis using principal-axis factoring with Promax rotation identified five empirical groupings. These are reported as provisional item clusters rather than confirmed latent constructs: (1) Technological and Infrastructural Barriers; (2) Training and Implementation-Adaptation Indicators; (3) Workflow and Relational Barriers; (4) Governance, Legal, and Policy Barriers; and (5) Perceived Usefulness. The second grouping was conceptually heterogeneous: it combined an item on insufficient personal training, an item on continuous staff training, an item on digital-skill barriers, and an item on the need to change traditional healthcare delivery. For this reason, it is not interpreted as a unitary “barrier” construct, and the constituent items remain central to substantive interpretation.

Most items showed their strongest loading on the theoretically expected factor. However, the item “Telemedicine requires changes in traditional healthcare delivery” showed stronger empirical alignment with the Training and Implementation-Adaptation Indicators factor and was therefore included in this domain. Conversely, the item “Not all healthcare services are suitable for telemedicine” showed a less specific factorial pattern and was retained for item-level descriptive interpretation but excluded from the revised composite domain scores.

Internal consistency was recalculated for the provisional groupings. Alpha was 0.791 for Technological and Infrastructural Barriers, 0.723 for the Training and Implementation-Adaptation Indicators grouping, 0.725 for Workflow and Relational Barriers, 0.886 for Governance, Legal, and Policy Barriers, and 0.832 for Perceived Usefulness. These coefficients support internal consistency of the observed item sets but do not establish factorial validity. Composite scores were therefore used only for exploratory association analyses.

Thus, all five revised multi-item domains demonstrated Cronbach’s alpha coefficients above the predefined 0.70 threshold. Composite scores based on this empirically supported and theoretically interpretable structure were therefore used in the subsequent correlation and multivariable analyses (Table 2).

### 3.3. Perceived Barriers to Telemedicine Implementation

Participants reported several barriers to telemedicine implementation. The strongest barrier was related to training and digital competence. Most participants agreed or strongly agreed that healthcare professionals need continuous training on telemedicine (84.7%), and 64.7% agreed that lack of digital skills represents a barrier to implementation. More than half of the samples (53.2%) reported insufficient training for telemedicine use.

Governance and policy-related issues were also prominent. A lack of clear protocols and guidelines was reported by 61.5% of participants, while unclear or insufficient reimbursement was reported by 50.3%. Similarly, 50.3% perceived insufficient institutional and managerial support, and 47.2% agreed that legal responsibility in telemedicine services is unclear.

Technological barriers were present but less dominant than educational and governance-related barriers. Data security and protection of patient information was the most frequently endorsed technological concern, reported by 52.0% of participants. Technical problems were reported by 46.2%, lack of integration between existing information systems and telemedicine platforms by 40.2%, insufficient technological infrastructure by 38.4%, and inadequate technological devices by 31.1%.

Workflow and relational barriers showed more variable levels of agreement. While 65.8% of participants agreed that telemedicine requires changes in the traditional way of delivering healthcare, fewer participants perceived telemedicine as increasing workload (30.5%), being difficult to integrate into daily routines (26.8%), or limiting the healthcare professional–patient relationship (25.9%). However, 55.3% agreed that not all healthcare services are suitable for telemedicine (Table 3).

### 3.4. Perceived Usefulness and Future Willingness to Use Telemedicine

Overall, participants expressed a moderately positive perception of telemedicine usefulness. More than half agreed that telemedicine can improve access to healthcare services (57.5%), support more efficient use of time and resources (61.9%), improve quality of care (60.4%), and be particularly useful for rural or remote areas (62.3%). Future willingness to use telemedicine was also relatively high, with 817 participants (67.4%) agreeing or strongly agreeing that they would be willing to use telemedicine in future practice.

Participants with previous telemedicine experience reported higher willingness to use telemedicine in the future than those without previous experience. Among participants with prior experience, the mean willingness score was 3.86 (SD = 1.05), and 73.5% reported agreement or strong agreement. Among those without prior experience, the mean willingness score was 3.62 (SD = 1.07), and 63.9% reported agreement or strong agreement (Table 4).

### 3.5. Associations Between Implementation Domains and Future Willingness

Spearman’s correlations were calculated to explore associations between implementation domains and willingness to use telemedicine in future practice. Perceived usefulness showed the strongest association with future willingness (ρ = 0.625, *p* < 0.001). Professional confidence was also positively associated with future willingness (ρ = 0.353, *p* < 0.001). Training and digital competence barriers (ρ = 0.163, *p* < 0.001), governance/policy barriers (ρ = 0.142, *p* < 0.001), and technological barriers (ρ = 0.122, *p* < 0.001) showed weaker positive correlations. Workflow and relational barriers showed a weak negative association with future willingness (ρ = −0.069, *p* = 0.017) (Table 5).

These results suggest that professionals’ future willingness to use telemedicine is primarily driven by perceived usefulness and confidence, whereas perceived workflow burden and relational barriers may reduce willingness.

### 3.6. Multivariable Linear Regression Predicting Future Willingness to Use Telemedicine

A multivariable linear regression model was conducted using the refined questionnaire domain scores derived from the dimensionality analysis. The dependent variable was future willingness to use telemedicine, measured on a five-point scale. The model included previous telemedicine experience, perceived usefulness, professional confidence, the four implementation-barrier composite domains, and relevant professional and sociodemographic characteristics.

The model explained 45.9% of the variance in future willingness to use telemedicine (R^2^ = 0.459; adjusted R^2^ = 0.451). Perceived usefulness showed the strongest adjusted cross-sectional association with willingness (B = 0.665, 95% CI 0.602–0.727, *p* < 0.001). Professional confidence was also positively associated with willingness (B = 0.114, 95% CI 0.065–0.162, *p* < 0.001). These coefficients are reported consistently throughout the Abstract, Results, Discussion, and Conclusions.

Perceived usefulness remained the strongest independent predictor of future willingness (B = 0.665, 95% CI 0.602–0.727, *p* < 0.001). Professional confidence was also independently associated with greater willingness (B = 0.114, 95% CI 0.065–0.162, *p* < 0.001).

Workflow and relational barriers showed an independent negative association with future willingness (B = −0.138, 95% CI −0.187 to −0.088, *p* < 0.001), indicating that greater perceived disruption of clinical workflows and professional–patient relationships was associated with lower willingness to use telemedicine.

Technological and infrastructural barriers, training/digital competence and implementation adaptation, and governance/legal/policy barriers were not independently associated with future willingness after adjustment. Previous telemedicine experience was also no longer statistically significant.

Compared with nurses, participants belonging to other healthcare professional groups showed slightly greater future willingness (B = 0.179, 95% CI 0.065–0.294, *p* = 0.002), whereas physicians did not significantly differ from nurses. No statistically significant independent associations were observed for level of care, years of professional experience, or educational level (Table 6).

### 3.7. Sensitivity Analysis: Multivariable Logistic Regression Predicting High Future Willingness

A multivariable logistic regression analysis was conducted as a sensitivity analysis using the refined questionnaire domain scores. High future willingness was defined as agreement or strong agreement with the statement “I am willing to use telemedicine in my future practice.”

The findings were consistent with those obtained from the primary linear regression model. Perceived usefulness remained the strongest independent predictor of high future willingness. For each one-point increase in perceived usefulness, the odds of reporting high willingness increased more than fivefold (OR = 5.41, 95% CI 4.32–6.79, *p* < 0.001). Professional confidence was also positively associated with high willingness (OR = 1.42, 95% CI 1.22–1.65, *p* < 0.001).

Conversely, greater workflow and relational barriers were independently associated with lower odds of high future willingness (OR = 0.59, 95% CI 0.48–0.71, *p* < 0.001).

Technological and infrastructural barriers, training/digital competence and implementation adaptation, governance/legal/policy barriers, and previous telemedicine experience were not independently associated with high willingness after adjustment.

Among the professional and contextual characteristics, participants working in secondary care had lower odds of high willingness than those working in primary care (OR = 0.66, 95% CI 0.44–0.99, *p* = 0.042). Participants holding a Master of Science degree had higher odds of high willingness than those with a Bachelor degree (OR = 1.69, 95% CI 1.11–2.56, *p* = 0.014). No statistically significant independent associations were observed for profession, tertiary or other care settings, years of professional experience, professional master’s degree, or doctorate (Table 7).

### 3.8. Open-Ended Responses and Co-Design Priorities

The open-ended responses provided supplementary descriptive context. Content-oriented coding identified recurrent mentions of technological infrastructure/equipment, training and digital competence, legal/regulatory clarity, protocols/guidelines, financial or reimbursement issues, institutional support, data security, resistance or acceptance of change, and patient access/awareness. Because categories were non-mutually exclusive, these frequencies should be interpreted as coded mentions rather than population prevalence estimates or formal qualitative theme prevalence.

Of the 1213 survey participants, 1206 provided a substantive response to the barrier prompt and 1206 provided a substantive response to the suggested-intervention prompt. Accordingly, percentages in Table 8 are calculated separately using *n* = 1206 as the denominator for each prompt.

When participants were asked which measures or interventions would most support effective telemedicine implementation, training and digital competence emerged as the most frequently mentioned priority, followed by improvement of technological infrastructure and equipment. Other recurring priorities included legal and regulatory clarification, development of protocols and guidelines, institutional and managerial support, patient and professional awareness, financial sustainability, and data security (Table 8).

### 3.9. Integration of Quantitative and Open-Ended Survey Findings Within the Living Lab-Informed Framework

Integration of the structured survey and open-ended-response mapping provides a descriptive bridge from readiness assessment toward possible future co-design. The quantitative findings identify perceived usefulness, professional confidence, workflow/relational concerns, and frequently endorsed implementation issues; the open-ended responses add participant-generated suggestions concerning training, infrastructure, governance, protocols, institutional support, and patient engagement. These convergent signals are interpreted as preliminary topics for subsequent stakeholder consultation, not as validated national priorities.

The open-ended-response mapping was directionally consistent with the structured survey in identifying multiple perceived implementation needs, including infrastructure, training, legal clarity, protocols, institutional support, financial sustainability, data security, and patient/professional engagement. This convergence supports their use as topics for subsequent stakeholder consultation; it does not establish nationally representative priorities or demonstrate that co-design has already occurred.

Taken together, the findings suggest three broad areas that could be taken forward for further stakeholder deliberation: professional capability and training; technological infrastructure and interoperability; and governance/organizational arrangements, including protocols, legal responsibility, reimbursement, and institutional support. Because the sample was non-probabilistic and heavily weighted toward nurses and primary care, these areas should be treated as priorities emerging from this survey sample rather than as definitive priorities for the Albanian healthcare system (Figure 2).

## 4. Discussion

This study explored healthcare professionals’ perceptions of telemedicine implementation in Albania through a cross-sectional, Living Lab-informed readiness-assessment perspective. The findings should be interpreted as perceptions of the 1213 respondents rather than as estimates of national readiness. The study identified strong endorsement of training and adaptation needs, moderate-to-positive perceived usefulness, and a 67.4% proportion reporting high future willingness. Multivariable models showed cross-sectional associations between willingness, perceived usefulness, professional confidence, and workflow/relational concerns; they do not establish causal predictors of future adoption.

These findings are consistent with international evidence indicating that telemedicine adoption cannot be understood solely as a matter of technological availability. Sustainable implementation also depends on professional preparedness, compatibility with clinical workflows, organizational support, governance arrangements, financing, privacy and data protection, and the perceived value of digital care for patients and professionals [28,29,30,31,32,33]. Accordingly, the present findings should be interpreted as identifying priorities that may warrant further consideration and investigation rather than as providing definitive recommendations for telemedicine implementation at the national level.

### 4.1. Professional Readiness, Perceived Usefulness, and Implementation Barriers

Perceived usefulness showed the strongest adjusted statistical association with future willingness (linear model B = 0.665; logistic sensitivity model OR = 5.41), while professional confidence was positively associated and workflow/relational barriers were negatively associated. Because all measures were collected cross-sectionally in the same survey, these associations are non-causal and may partly reflect shared-method variance.

These findings are consistent with technology-acceptance literature indicating that professionals are more receptive to digital technologies when they perceive clear clinical and organizational benefits and feel capable of incorporating them into practice [30,33]. In healthcare settings, usefulness is likely to be linked to the extent to which a technology addresses meaningful clinical or organizational needs, such as access to specialist consultation, chronic disease follow-up, continuity after discharge, patient education, remote triage, or support for geographically underserved populations.

The strong association between perceived usefulness and future willingness should, however, be interpreted cautiously. The two constructs are conceptually proximal and were assessed using the same questionnaire at the same time point. Consequently, part of the observed association may reflect shared-method variance, response consistency, or partial construct overlap. Furthermore, because the study was cross-sectional, the statistical role of perceived usefulness as a predictor within the regression model should not be interpreted as evidence of temporal precedence or causality.

Workflow and relational barriers were negatively associated with future willingness. Participants appeared less willing to use telemedicine when they perceived it as potentially increasing workload, disrupting daily routines, complicating integration with existing care processes, or weakening the professional–patient relationship. Similar concerns have been identified in previous digital-health research, particularly in relation to duplicated documentation, fragmented information systems, reduced interpersonal communication, and additional organizational demands [29,30,31].

These findings suggest that future telemedicine initiatives may benefit from attention to workflow integration, scheduling, documentation, referral pathways, professional responsibilities, communication practices, and continuity between remote and face-to-face care. Hybrid models may be particularly relevant, allowing telemedicine to complement rather than replace in-person services where physical assessment or direct interpersonal interaction remains necessary.

### 4.2. Training, Digital Competence, Governance, and Organizational Readiness

Training and digital competence emerged as prominent item-level concerns in both the structured and open-ended responses. Importantly, the exploratory factor grouping that combined training need, digital-skill deficits, and adaptation of traditional healthcare delivery should not be treated as a single validated barrier construct. Its nonsignificant adjusted association with willingness further supports a cautious, item-level interpretation. The descriptive findings nevertheless indicate that participants frequently perceived continuous training and digital competence as important implementation needs.

Training for telemedicine may need to extend beyond the technical operation of digital platforms. Areas for consideration include remote clinical assessment, patient selection, virtual communication, documentation, privacy and data protection, clinical risk, legal responsibilities, escalation procedures, and recognition of situations requiring face-to-face assessment. Simulation, supervised practice, mentoring, peer support, and pilot implementation may also provide opportunities to strengthen professional confidence before wider use.

Governance-related concerns were similarly prominent. Participants identified unclear protocols, legal responsibilities, reimbursement mechanisms, and insufficient institutional support as important challenges. These findings are consistent with international literature emphasizing the importance of liability, consent, privacy, data governance, financing, quality standards, and organizational accountability in telemedicine implementation [34,35,36].

Rather than supporting definitive policy prescriptions, these findings suggest several areas that may merit consideration in future planning and stakeholder consultation. These include development of clinical and operational guidance, clarification of professional responsibilities, appropriate reimbursement mechanisms, institutional technical support, interoperability with existing information systems, and organizational arrangements that prevent telemedicine from being experienced as additional or unrecognized workload.

The relatively moderate endorsement of some technological barriers compared with training and governance concerns is also noteworthy. This may suggest that, for many participants, the primary challenge is not simply access to technology but the institutional and professional conditions required to use telemedicine safely, consistently, and legitimately within routine care.

### 4.3. From Readiness Assessment Toward Living Lab-Informed Co-Design

The present study should be understood as an exploratory readiness-assessment phase informed by Living Lab principles rather than as a completed Living Lab or co-design intervention. Healthcare professionals provided survey-based information about perceived barriers, readiness indicators, and potential implementation priorities, but the study did not include iterative stakeholder workshops, shared design sessions, prototyping, testing, or implementation cycles. This distinction is essential when interpreting both the title and the contribution of the study.

Within this more limited scope, readiness assessment may provide a useful foundation for subsequent participatory activities. Professional perceptions identified in the present study may help inform future discussions concerning training, workflow redesign, governance, legal support, technological infrastructure, and relational quality.

The open-ended survey questions should similarly be interpreted as providing supplementary contextual information rather than constituting an independent qualitative or formal mixed-methods component. Their contribution was to identify recurrent issues and participant-generated priorities that complemented the structured survey findings.

A subsequent Living Lab phase would require broader stakeholder participation, including healthcare professionals, patients, caregivers, managers, policymakers, legal experts, technology developers, and information-technology professionals. Future activities could include identification of priority use cases, participatory design of workflows and protocols, development of prototypes, pilot implementation, iterative feedback, and evaluation of feasibility, acceptability, safety, workload, patient experience, equity, costs, and sustainability.

In this sense, the present findings may help provide empirical input for moving toward participatory co-design, but they should not be interpreted as evidence that co-design itself was undertaken in this study.

### 4.4. Implications for Practice, Education, Leadership, and Policy Consideration

The findings may have implications for professional education, organizational planning, and future telemedicine development, but they do not justify direct national policy prescriptions. Given the predominance of nurses (73.5%) and primary-care professionals (62.5%), the aggregate results are especially influenced by these groups’ operational perspectives. Smaller professional subgroups, including physicians, are not represented with comparable precision.

Given the predominance of nurses in the sample, the results particularly highlight areas that may be relevant to nursing education and leadership. Nurses frequently contribute to chronic disease management, patient education, follow-up, care coordination, and continuity of care, all of which may involve telemedicine in future service models. Educational programs may therefore consider competencies related to remote assessment, virtual communication, documentation, privacy, clinical decision-making, patient safety, and criteria for escalation to in-person care.

Nurse leaders and other healthcare managers may also have an important role in supporting training, workflow redesign, protocol development, implementation evaluation, and coordination between clinical teams, organizational leadership, technical specialists, and other stakeholders. However, because the sample was not nationally representative, these implications should be interpreted as areas for consideration rather than as population-level conclusions about the Albanian nursing workforce.

At a broader system level, the findings suggest several potential priorities for further evaluation, including professional training, clear governance mechanisms, interoperability, legal and regulatory clarification, organizational support, sustainable financing, and attention to equitable access. These findings may inform future stakeholder consultation or pilot initiatives but are not sufficient, on their own, to justify definitive national policy prescriptions.

Telemedicine may offer particular opportunities in rural or underserved settings, but attention is also needed to potential digital inequalities. Patients with limited connectivity, digital literacy, access to devices, privacy, or social support may experience barriers to remote care. Future implementation planning may therefore consider accessible technologies, digital assistance, alternative communication channels, and continued availability of face-to-face services.

Rather than recommending immediate large-scale implementation, the current findings support the value of carefully selected pilot projects in real-world settings. Such pilots could evaluate locally relevant telemedicine use cases and generate evidence regarding feasibility, safety, professional workload, patient experience, organizational impact, equity, and sustainability before decisions about wider scaling are considered.

### 4.5. Strengths and Limitations

This study has several strengths. It included a large sample of healthcare professionals, examined multiple implementation dimensions, combined structured items with supplementary open-ended responses, and used both linear and logistic multivariable models. The Living Lab-informed framing also provides a practical bridge between readiness assessment and future participatory development. At the same time, the newly developed questionnaire remains exploratory and the open-ended component was designed for contextual mapping rather than as an independent qualitative study.

An additional reporting limitation concerns duplicate-response verification. Because the analytical dataset was anonymous and did not retain direct identifiers, four pairs of identical response profiles with near-adjacent timestamps could not be definitively classified as duplicate participation. They were retained in the primary analysis, while a sensitivity check excluding the later record in each pair showed negligible changes in key descriptive estimates. The questionnaire also did not undergo a separately documented formal pilot study before field administration; its measurement properties therefore remain exploratory despite the full-sample EFA and internal-consistency analysis.

Several limitations should nevertheless be considered. First, the cross-sectional design precludes causal and temporal inference. Terms such as “predictor” in statistical model descriptions refer to model covariates and should not be interpreted as evidence that the measured factors predict subsequent telemedicine adoption.

Second, participants were recruited using a voluntary, non-probability strategy through institutional channels and professional networks. This creates potential selection and self-selection bias: professionals with greater digital literacy, stronger interest in telemedicine, or more positive or negative pre-existing views may have been more likely to respond. The exposed population was unknown and a valid response rate could not be calculated.

The predominance of nurses (73.5%) and primary-care professionals (62.5%) means that overall descriptive estimates are weighted toward these groups. Regional information was available for only 770 participants. Accordingly, the findings should not be interpreted as nationally representative estimates of Albanian healthcare professionals or as healthcare-system-wide priorities.

Third, the questionnaire was developed specifically for this study. The factor solution and composite scores are exploratory and were derived from the same dataset used for the substantive association analyses. No independent CFA, convergent/discriminant validation, test–retest assessment, or measurement-invariance testing was performed. The item grouping that combines training, digital competence, and implementation adaptation is conceptually heterogeneous and should not be interpreted as an established latent construct. These limitations are why item-level findings and cautious wording are emphasized throughout the revised manuscript.

Two questionnaire items showed less specific factorial behavior and were excluded from the revised composite scores while being retained for item-level interpretation. In addition, professional confidence and future willingness were assessed using single items. Future instrument-development research should consider multi-item measurement of these constructs and further refinement of items demonstrating weaker factorial specificity.

Fourth, all quantitative data were self-reported and may be influenced by social-desirability, recall, and response-style effects. Of particular relevance, perceived usefulness and future willingness were measured through the same questionnaire and at the same time point. Their strong association may therefore partly reflect shared-method variance and conceptual proximity in addition to a substantive relationship.

Fifth, some variables, particularly age, required data cleaning because respondents occasionally entered year of birth or other non-standard values.

Sixth, the two open-ended questions were supplementary survey items. Content-oriented coding was conducted by two coders using NVivo 15.6.0 (Lumivero) and provided useful descriptive context; however, this component does not have the depth of an interview- or focus-group-based qualitative study. Categories were not mutually exclusive, and no formal intercoder-reliability coefficient was calculated. Accordingly, the open-ended findings should be interpreted as supplementary content mapping rather than as an independent qualitative investigation.

Finally, the study focused exclusively on healthcare professionals and did not include patients, caregivers, managers, policymakers, legal experts, or technology developers. The findings therefore reflect only one stakeholder perspective and represent an initial readiness-assessment phase rather than a complete Living Lab process.

### 4.6. Future Research

Future research should extend the present findings through more representative, longitudinal, psychometric, and participatory designs.

First, the questionnaire requires dedicated psychometric validation in new data. Ideally, a future study should use a prespecified split-sample or, preferably, an independent sample for EFA/CFA, followed by assessment of convergent and discriminant validity, test–retest reliability, and measurement invariance across professions and care settings. Particular attention should be given to separating training need, competence deficits, and implementation adaptation rather than combining them automatically into one score.

Second, longitudinal studies should examine whether perceived usefulness, confidence, training, organizational support, and implementation barriers are associated with subsequent telemedicine uptake and sustained use. Objective indicators—such as platform login rates, completed teleconsultations, referral completion, documentation burden, response times, and continued use over time—would reduce reliance on same-survey self-report and help address shared-method variance.

Third, future research should incorporate a broader range of stakeholders, including patients, caregivers, healthcare managers, policymakers, legal experts, professional organizations, and technology providers. Interviews, focus groups, and participatory workshops may provide greater insight into professional and patient experiences than open-ended survey questions alone.

Finally, Living Lab-informed research could progress toward genuine co-design by identifying priority use cases, developing workflows and protocols collaboratively, testing prototype services in selected settings, and evaluating feasibility, acceptability, safety, equity, workload, patient experience, costs, and sustainability before consideration of broader implementation.

Overall, the present study provides exploratory evidence regarding healthcare professionals’ perceptions of telemedicine implementation in Albania. The findings may inform the identification of priorities for further investigation, stakeholder engagement, and future participatory development, but broader evidence will be required before population-level or national policy conclusions can be drawn.

## 5. Conclusions

This cross-sectional study explored healthcare professionals’ perceptions of telemedicine implementation in Albania using a Living Lab-informed readiness-assessment perspective. The results describe the views of a large but non-probability sample that was predominantly nursing- and primary-care-based. They should not be interpreted as national estimates of telemedicine readiness.

Perceived usefulness showed the strongest independent statistical association with future willingness to use telemedicine, while professional confidence was also positively associated with willingness. Conversely, workflow and relational barriers were associated with lower willingness. Given the cross-sectional design and the conceptual proximity between perceived usefulness and future willingness, these associations should not be interpreted as causal.

The findings suggest areas for further investigation and stakeholder consultation, including professional training, workflow integration, technological infrastructure, governance, legal/regulatory clarity, institutional support, and equitable access. The questionnaire domains remain exploratory, and the open-ended findings represent supplementary content mapping. Future confirmatory psychometric work and genuine participatory Living Lab activities are needed before stronger implementation or policy conclusions are warranted.

Rather, the findings provide preliminary empirical evidence that may inform further stakeholder consultation, participatory co-design, and pilot implementation. Future Living Lab activities should include a broader range of stakeholders and incorporate iterative co-design, prototyping, testing, and evaluation before wider implementation is considered. Further representative, longitudinal, and confirmatory psychometric research is also needed to strengthen the evidence base for telemedicine implementation in the Albanian healthcare context.

## Figures and Tables

**Figure 1:**
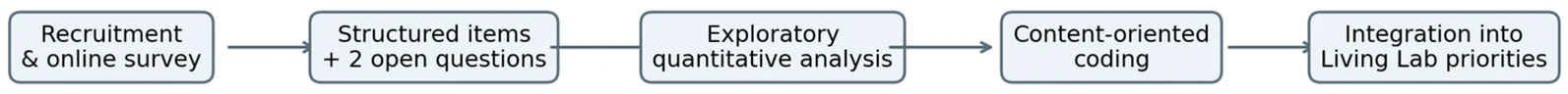
Methodological workflow of the cross-sectional Living Lab-informed readiness-assessment study.

**Figure 2:**
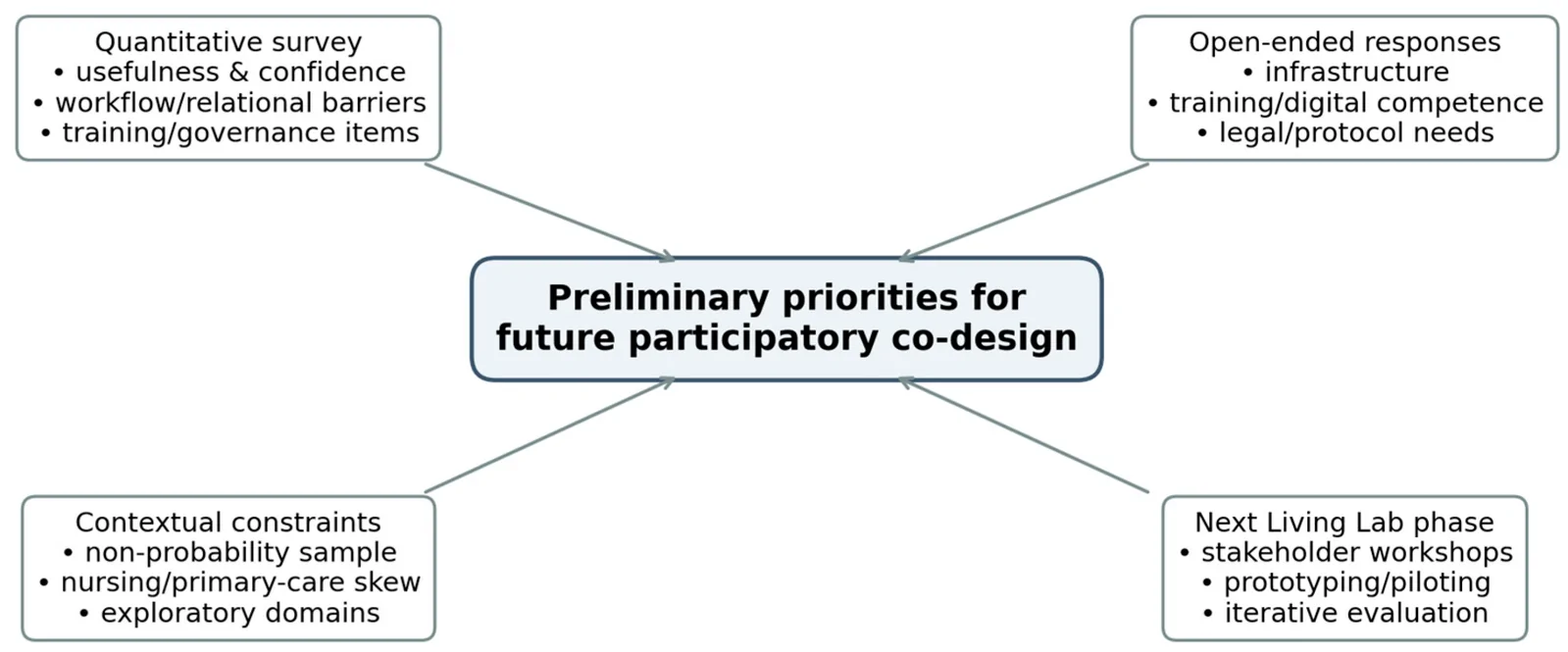
Integration of structured survey findings and open-ended-response mapping within the proposed transition from readiness assessment to future Living Lab co-design.

**Table 1:** Participants’ characteristics.

Variable	Category	*n*	%
Profession	Nurse	892	73.5
	Physician	68	5.6
	Midwife	60	4.9
	Laboratory technician	62	5.1
	Imaging technician	75	6.2
	Physiotherapist	27	2.2
	Speech therapist	7	0.6
	Other	22	1.8
Educational level	Bachelor	526	43.4
	Professional master	335	27.6
	Master of Science	328	27.0
	Doctorate	24	2.0
Years of professional experience	<5 years	326	26.9
	5–10 years	255	21.0
	11–20 years	313	25.8
	>20 years	318	26.2
	Missing	1	0.1
Level of care	Primary	758	62.5
	Secondary	256	21.1
	Tertiary	86	7.1
	Other	113	9.3
Previous telemedicine experience	Yes	449	37.0
	No	764	63.0

**Table 2:** Exploratory factor structure and internal consistency of the questionnaire domains.

Questionnaire Item	F1 Technological Barriers	F2 Training & Implementation-Adaptation Indicators	F3 Workflow & Relational Barriers	F4 Governance, Legal & Policy Barriers	F5 Perceived Usefulness	Communality
Technological infrastructure is insufficient for telemedicine implementation	0.621	0.008	−0.033	0.041	0.010	0.404
Technological devices are not adequate for telemedicine services	0.820	−0.150	−0.015	−0.019	0.011	0.526
Technical problems hinder regular telemedicine use	0.671	0.043	0.097	−0.054	0.046	0.522
Existing information systems are not integrated with telemedicine platforms	0.702	0.003	−0.060	0.110	−0.028	0.545
Data security and protection are major concerns †	0.262	0.260	0.096	−0.079	0.033	0.226
I have not received sufficient training for telemedicine use	0.092	0.492	0.001	0.112	−0.132	0.365
Healthcare staff need continuous telemedicine training	−0.163	0.805	−0.072	−0.054	0.025	0.466
Lack of digital skills is a barrier to telemedicine implementation	0.056	0.713	0.010	−0.038	−0.043	0.511
Telemedicine requires changes in traditional healthcare delivery	0.022	0.542	0.009	0.056	0.101	0.405
Telemedicine increases healthcare staff workload	−0.055	−0.028	0.771	−0.076	0.048	0.485
Integrating telemedicine into daily work routines is difficult	0.025	−0.020	0.853	−0.102	0.022	0.642
Telemedicine limits the healthcare professional–patient relationship	0.033	−0.092	0.500	0.186	−0.100	0.383
Not all healthcare services are suitable for telemedicine †	−0.069	0.077	0.240	0.351	−0.047	0.284
Clear protocols and guidelines for telemedicine are lacking	−0.041	0.208	0.046	0.578	−0.067	0.516
The current legal framework does not sufficiently support telemedicine	−0.014	−0.017	0.024	0.697	0.019	0.483
Clear health policies for telemedicine integration are lacking	−0.026	−0.033	−0.051	0.860	0.012	0.640
Financial reimbursement is unclear or insufficient	0.022	−0.067	−0.066	0.839	0.051	0.614
Legal responsibility in telemedicine services is unclear	0.004	−0.036	−0.040	0.839	−0.009	0.633
Institutional and managerial support is insufficient	0.083	−0.035	−0.062	0.753	0.034	0.564
Telemedicine improves patient access to healthcare services	−0.014	0.010	0.013	0.013	0.788	0.625
Telemedicine supports more efficient use of time and resources	0.037	−0.052	−0.003	0.058	0.849	0.716
Telemedicine may improve quality of healthcare	0.009	0.018	0.033	−0.050	0.792	0.624
Telemedicine is particularly useful for rural or remote areas	−0.045	0.075	0.019	0.070	0.547	0.342
Cronbach’s α for retained composite	0.791	0.723	0.725	0.886	0.832	—
Items retained in composite, *n*	4	4	3	6	4	—

Note. Exploratory factor analysis was conducted on 23 Likert-scale items using principal-axis factoring with Promax oblique rotation. Sampling adequacy was very good (KMO = 0.905), and Bartlett’s test of sphericity was significant, χ^2^(253) = 11,061.66, *p* < 0.001. Parallel analysis supported retention of five exploratory factors. Loadings ≥ 0.40 are shown in bold. † The dagger denotes items retained for item-level descriptive reporting but excluded from composite scores because they did not demonstrate a sufficiently specific association with a single grouping. The F2 label is deliberately descriptive because its items combine training need, skill deficit, and implementation adaptation and should not be interpreted as a single established latent barrier construct.

**Table 3:** Item-level descriptive results.

Item	Mean	SD	Agreement *n*	Agreement %
Technological infrastructure is insufficient for telemedicine implementation	2.97	1.23	466	38.4
Technological devices are not adequate for telemedicine services	2.60	1.34	377	31.1
Technical problems hinder regular telemedicine use	3.10	1.27	561	46.2
Existing information systems are not integrated with telemedicine platforms	3.00	1.21	488	40.2
Data security and protection are major concerns	3.26	1.34	631	52.0
I have not received sufficient training for telemedicine use	3.22	1.27	645	53.2
Healthcare staff need continuous telemedicine training	4.12	0.92	1027	84.7
Lack of digital skills is a barrier to telemedicine implementation	3.60	1.15	785	64.7
Telemedicine requires changes in traditional healthcare delivery	3.62	1.04	798	65.8
I feel professionally confident using telemedicine in clinical practice	3.16	1.14	535	44.1
Telemedicine increases healthcare staff workload	2.69	1.27	370	30.5
Integrating telemedicine into daily work routines is difficult	2.62	1.17	325	26.8
Telemedicine limits the healthcare professional–patient relationship	2.53	1.26	314	25.9
Not all healthcare services are suitable for telemedicine	3.36	1.18	671	55.3
Clear protocols and guidelines for telemedicine are lacking	3.54	1.16	746	61.5
The current legal framework does not sufficiently support telemedicine	3.07	1.04	417	34.4
Clear health policies for telemedicine integration are lacking	3.27	1.08	563	46.4
Financial reimbursement is unclear or insufficient	3.41	1.05	610	50.3
Legal responsibility in telemedicine services is unclear	3.27	1.10	573	47.2
Institutional and managerial support is insufficient	3.34	1.13	610	50.3
Telemedicine improves patient access to healthcare services	3.45	1.07	697	57.5
Telemedicine supports more efficient use of time and resources	3.53	1.06	751	61.9
Telemedicine may improve quality of healthcare	3.52	1.09	733	60.4
Telemedicine is particularly useful for rural or remote areas	3.56	1.20	756	62.3
I am willing to use telemedicine in my future practice	3.71	1.07	817	67.4

Note. Agreement was defined as responses of “agree” or “strongly agree”.

**Table 4:** Future willingness to use telemedicine by previous telemedicine experience.

Previous Telemedicine Experience	*n*	Mean Willingness	SD	Agreement %
Yes	449	3.86	1.05	73.5
No	764	3.62	1.07	63.9

**Table 5:** Correlations between implementation domains and future willingness to use telemedicine.

Domain	Spearman’s ρ	*p*-Value
Perceived usefulness	0.625	<0.001
Professional confidence	0.353	<0.001
Training and digital competence barriers	0.163	<0.001
Governance, legal, and policy barriers	0.142	<0.001
Technological barriers	0.122	<0.001
Workflow and relational barriers	−0.069	0.017

**Table 6:** Multivariable linear regression predicting future willingness to use telemedicine using the refined questionnaire domains.

Predictor	B	95% CI	*p*-Value
Previous telemedicine experience	0.056	−0.042 to 0.154	0.261
Perceived usefulness	0.665	0.602 to 0.727	<0.001
Professional confidence	0.114	0.065 to 0.162	<0.001
Technological and infrastructural barriers	0.015	−0.040 to 0.069	0.596
Training & implementation-adaptation indicators	0.050	−0.024 to 0.124	0.188
Workflow and relational barriers	−0.138	−0.187 to −0.088	<0.001
Governance, legal, and policy barriers	0.065	−0.003 to 0.133	0.063
Physician vs. nurse	−0.037	−0.250 to 0.176	0.732
Other healthcare professional vs. nurse	0.179	0.065 to 0.294	0.002
Secondary care vs. primary care	−0.041	−0.157 to 0.075	0.486
Tertiary care vs. primary care	0.058	−0.126 to 0.241	0.537
Other care setting vs. primary care	−0.117	−0.310 to 0.077	0.237
5–10 years vs. <5 years experience	−0.087	−0.220 to 0.046	0.200
11–20 years vs. <5 years experience	−0.107	−0.235 to 0.021	0.101
>20 years vs. <5 years experience	−0.091	−0.218 to 0.036	0.159
Professional master vs. Bachelor	0.046	−0.068 to 0.160	0.433
Master of Science vs. Bachelor	0.089	−0.030 to 0.208	0.143
Doctorate vs. Bachelor	0.386	−0.113 to 0.884	0.129

*Note. n = 1212. The dependent variable was willingness to use telemedicine in future practice, scored from 1 to 5. Composite scores were calculated according to the refined five-factor structure identified through exploratory factor analysis. Professional confidence was retained as a separate single-item predictor. Robust HC3 standard errors were used. Reference categories were nurse, primary care, <5 years of professional experience, and Bachelor degree. R*^2^ *= 0.459; adjusted R*^2^ *= 0.451. CI = confidence interval.*

**Table 7:** Multivariable logistic regression predicting high future willingness to use telemedicine using the refined questionnaire domains.

Predictor	OR	95% CI	*p*-Value
Previous telemedicine experience	1.15	0.82 to 1.62	0.427
**Perceived usefulness**	**5.41**	**4.32 to 6.79**	**<0.001**
**Professional confidence**	**1.42**	**1.22 to 1.65**	**<0.001**
Technological and infrastructural barriers	1.15	0.95 to 1.40	0.159
Training & implementation-adaptation indicators	1.14	0.90 to 1.45	0.275
**Workflow and relational barriers**	**0.59**	**0.48 to 0.71**	**<0.001**
Governance, legal, and policy barriers	1.20	0.94 to 1.53	0.150
Physician vs. nurse	1.48	0.63 to 3.47	0.370
Other healthcare professional vs. nurse	1.32	0.88 to 1.99	0.177
**Secondary care vs. primary care**	**0.66**	**0.44 to 0.99**	**0.042**
Tertiary care vs. primary care	0.66	0.34 to 1.29	0.223
Other care setting vs. primary care	0.65	0.37 to 1.15	0.142
5–10 years vs. <5 years experience	0.85	0.54 to 1.34	0.490
11–20 years vs. <5 years experience	0.86	0.55 to 1.32	0.482
>20 years vs. <5 years experience	0.98	0.63 to 1.53	0.944
Professional master vs. Bachelor	1.43	0.97 to 2.10	0.067
**Master of Science vs. Bachelor**	**1.69**	**1.11 to 2.56**	**0.014**
Doctorate vs. Bachelor	2.05	0.54 to 7.77	0.290

*Note. n = 1212. High future willingness was defined as responding “agree” or “strongly agree” to the item “I am willing to use telemedicine in my future practice.” Composite predictors were calculated according to the refined five-factor structure identified through exploratory factor analysis. Reference categories were nurse, primary care, <5 years of professional experience, and Bachelor degree. OR = odds ratio; CI = confidence interval. Nagelkerke pseudo-R*^2^ *≈ 0.34.*

**Table 8:** Thematic Mapping of Open-Ended Responses.

Theme	Barriers *n*	Barriers %	Suggested Interventions *n*	Suggested Interventions %
Technological infrastructure/equipment	389	32.3	261	21.6
Training and digital competence	251	20.8	421	34.9
Legal/regulatory clarity	78	6.5	105	8.7
Protocols/guidelines	41	3.4	73	6.1
Institutional/managerial support	60	5.0	96	8.0
Financial/reimbursement issues	69	5.7	56	4.6
Data security/privacy	32	2.7	46	3.8
Resistance/acceptance/change	54	4.5	41	3.4
Patient access/awareness	106	8.8	100	8.3

*Note. Frequencies represent content-oriented coded mentions in the supplementary open-ended responses. Categories were not mutually exclusive; one response could receive multiple codes. For the barriers prompt, 1206 participants provided a substantive response and percentages use n = 1206 as the denominator. For the suggested-interventions prompt, 1206 participants provided a substantive response and percentages likewise use n = 1206 as the denominator. Seven blank or non-substantive responses per prompt were excluded from coding. Percentages should not be interpreted as mutually exclusive prevalence estimates.*

## Data Availability

The data supporting the findings of this study are available from the corresponding author upon reasonable request. The dataset is not publicly available because it contains information collected from healthcare professionals and is subject to privacy and ethical restrictions.

## Supplementary Materials

The following supporting information can be downloaded at: https://www.mdpi.com/article/10.3390/nursrep16100350/s1, Supplementary Material File S1. Study Questionnaire: Telemedicine Implementation in Albania.

## Public Involvement Statement

No patients, members of the public, consumers, or caregivers were involved in the design, conduct, reporting, or dissemination plans of this research.

## Guidelines and Standards Statement

This manuscript was prepared in accordance with the Strengthening the Reporting of Observational Studies in Epidemiology (STROBE) statement for cross-sectional studies.

## Use of Artificial Intelligence

During the preparation of this manuscript, the authors used OpenAI ChatGPT 5.5 to support language editing, improve textual clarity, and assist with the organization of selected manuscript sections. The authors critically reviewed and edited all AI-assisted outputs and take full responsibility for the accuracy, integrity, and content of the published work. Artificial intelligence was not used for data collection, statistical analysis, interpretation of the study findings, or generation of research data.

## Abbreviations

The following abbreviations are used in this manuscript:

**Abbreviation**	**Definition**
CFIR	Consolidated Framework for Implementation Research
CI	Confidence Interval
IBM SPSS	International Business Machines Statistical Package for the Social Sciences
OR	Odds Ratio
SD	Standard Deviation
STROBE	Strengthening the Reporting of Observational Studies in Epidemiology
WHO	World Health Organization

## References

[1] Erku D., Khatri R., Endalamaw A., Wolka E., Nigatu F., Zewdie A., Assefa Y. (2023). Digital health interventions to improve access to and quality of primary health care services: A scoping review. Int. J. Environ. Res. Public Health.

[2] Chaudhary U., Ali M.F., Kumar A., Sharma A., Jayakody D.K.N. (2025). Unleashing the power of wireless communication in healthcare by empowering patient care and connectivity: A comprehensive survey. IEEE Access.

[3] Kannadasan K. (2026). Multimodal AI and Telemedicine in Remote Health Monitoring. Multimodal AI in Healthcare: Fusion Intelligence for Advanced Health Diagnostics.

[4] Omboni S., Padwal R.S., Alessa T., Benczúr B., Green B.B., Hubbard I., Kario K., Khan N.A., Konradi A., Logan A.G. (2022). The worldwide impact of telemedicine during COVID-19: Current evidence and recommendations for the future. Connect. Health.

[5] Wittich L., Rödiger H., Rombey T., Brecher A.L., Kraft L., Sgraja S., Stein V., Henschke C. (2026). Navigating the complexities of digital health technology implementation: A scoping review of barriers and facilitators. Implement. Sci. Commun..

[6] Marinelli S., Basile G., Zaami S. (2022). Telemedicine, telepsychiatry and COVID-19 pandemic: Future prospects for global health. Healthcare.

[7] Doukani A., Cerga Pashoja A., Fanaj N., Qirjako G., Meksi A., Mustafa S., Vis C., Hug J. (2021). Organizational Readiness for Implementing an Internet-Based Cognitive Behavioral Therapy Intervention for Depression Across Community Mental Health Services in Albania and Kosovo: Directed Qualitative Content Analysis. JMIR Form. Res..

[8] World Health Organization (2024). Support tool to strengthen telemedicine: Resource for assessment, strategy development, and strengthening of telemedicine services. Support Tool to Strengthen Telemedicine: Resource for Assessment, Strategy Development, and Strengthening of Telemedicine Services.

[9] Anawade P.A., Sharma D., Gahane S., Anawade P.A., Sharma D.S. (2024). A comprehensive review on exploring the impact of telemedicine on healthcare accessibility. Cureus.

[10] Latifi R., Azevedo V., Boci A., Parsikia A., Latifi F., Merrell R.C. (2021). Telemedicine consultation as an indicator of local telemedicine champions’ contributions, health care system needs or both: Tales from two continents. Telemed. e-Health.

[11] Latifi R., Parsikia A., Boci A., Doarn C.R., Merrell R.C. (2020). Increased access to care through telemedicine in Albania: An analysis of 2724 patients. Telemed. e-Health.

[12] Marcolino M.S., Oliveira C.R.A.D., Sousa L.A.P.D., Nascimento B.R., Cimini C.C.R., Pessoa C.G., Vasconcelos-Santos D.V., Mancuzo E.V., Paixão G.M.d.M., Sanches G.T.d.S. (2026). The Telehealth Network of Minas Gerais, Brazil: Two decades of scaling and sustainability. BMJ Glob. Health.

[13] Duval J.S. (2022). Playful Health Technology: A Participatory, Research Through Design Approach to Applications for Wellness.

[14] Olldashi F., Latifi R., Parsikia A., Boci A., Qesteri O., Dasho E., Bakiu E. (2019). Telemedicine for neurotrauma prevents unnecessary transfers: An update from a nationwide program in Albania and analysis of 590 patients. World Neurosurg..

[15] Bode E., Shpuza A., Angjeli A. (2025). Telemedicine as a driver of efficiency in regional healthcare through service optimization in Albania. Arch. Balk. Med. Union.

[16] Otto L., Schlieter H., Harst L., Whitehouse D., Maeder A. (2023). The Telemedicine Community Readiness Model—Successful Telemedicine Implementation and Scale-Up. Front. Digit. Health.

[17] Weiner B.J. (2009). A Theory of Organizational Readiness for Change. Implement. Sci..

[18] Huang J.H., Iakovleva T.A., Bessant J. (2024). Fostering user involvement in collaborative innovation spaces: Insights from living labs. Int. J. Innov. Manag..

[19] Russo-Spena T., Salvatore C., Fairfield B., Giordano A., De Simone S., Illario M. (2025). Living Labs in digital health: A collaborative ecosystem approach for continuum of care. Front. Public Health.

[20] Okeke E.B., Forman D., Taylor E., Hyde E., Parente F., Kanmodi K.K., Wordsworth S. (2025). Barriers and facilitators to digital health adoption: A thematic analysis of healthcare professionals’ perspectives in rural Oyo State, Nigeria. BMC Digit. Health.

[21] Khan R., Khan S., Almohaimeed H.M., Almars A.I., Pari B. (2025). Utilization, challenges, and training needs of digital health technologies: Perspectives from healthcare professionals. Int. J. Med. Inform..

[22] Alsadaan N., Ramadan O.M.E. (2025). Barriers and facilitators in implementing evidence-based practice: A parallel cross-sectional mixed methods study among nursing administrators. BMC Nurs..

[23] Huo X., Qian Y., Siau K.L., Nah F.F.H. (2024). HCI in business and organizations: Digital transformation with HCI, metaverse, and AI technologies. Human-Computer Interaction in Various Application Domains.

[24] Papoutsi C., Wherton J., Shaw S., Morrison C., Greenhalgh T. (2021). Putting the social back into sociotechnical: Case studies of co-design in digital health. J. Am. Med. Inform. Assoc..

[25] Malloy J., Partridge S.R., Kemper J.A., Braakhuis A., Roy R. (2023). Co-design of digital health interventions with young people: A scoping review. Digit. Health.

[26] Ratnayake C., Antonie R.I., Hințea C., Forna M. (2025). Crafting the Future: How Starhaus Innovations Advance Sustainable Governance and Align with UN SDGs. Transylv. Rev. Adm. Sci..

[27] von Elm E., Altman D.G., Egger M., Pocock S.J., Gøtzsche P.C., Vandenbroucke J.P. (2007). The Strengthening the Reporting of Observational Studies in Epidemiology (STROBE) statement: Guidelines for reporting observational studies. Lancet.

[28] Saigí-Rubió F., Borges do Nascimento I., Robles N., Ivanovska K., Katz C., Azzopardi-Muscat N., Novillo-Ortiz D. (2022). The current status of telemedicine technology use across the World Health Organization European Region: An overview of systematic reviews. J. Med. Internet Res..

[29] Kruse C.S., Heinemann K., Moody L., Beck M., Rampersad A., Patel K. (2022). Facilitators and barriers to the adoption of telemedicine during the first year of COVID-19: Systematic review. J. Med. Internet Res..

[30] Gu Y., Guan Y., Meng Z. (2023). Health providers’ perceptions and experiences of using mHealth for chronic noncommunicable diseases: Qualitative systematic review and meta-synthesis. J. Med. Internet Res..

[31] Rettinger L., Kuhn S. (2023). Barriers to video call-based telehealth in allied health professions and nursing: Scoping review and mapping process. J. Med. Internet Res..

[32] World Health Organization (2021). Global Strategy on Digital Health 2020–2025.

[33] Harst L., Lantzsch H., Scheibe M. (2019). Theories predicting end-user acceptance of telemedicine use: Systematic review. J. Med. Internet Res..

[34] Sumner J., Tan S.Y., Wang Y., Keck C.H.S., Lee E.W.X., Chew E.H.H., Yip A.W. (2024). Co-designing remote patient monitoring technologies for inpatients: Systematic review. J. Med. Internet Res..

[35] Damschroder L.J., Reardon C.M., Widerquist M.A.O., Lowery J. (2022). The updated Consolidated Framework for Implementation Research based on user feedback. Implement. Sci..

[36] Greenhalgh T., Wherton J., Papoutsi C., Lynch J., Hughes G., A’Court C., Hinder S., Fahy N., Procter R., Shaw S. (2017). Beyond adoption: A new framework for theorizing and evaluating nonadoption, abandonment, and challenges to the scale-up, spread, and sustainability of health and care technologies. J. Med. Internet Res..

