# Supplementary material for: From Readiness Assessment to Co-Design: A Cross-Sectional, Living Lab-Informed Study of Telemedicine Implementation in Albania
Source: Nurs Rep. 2026 Sep 26;16(10):350. doi: 10.3390/nursrep16100350 (PMC13708037; doi:10.3390/nursrep16100350)
Supplement: Supplementary file 1 [file nursrep-16-00350-s001.zip › nursrep-4514607-supplementary.pdf]

Supplementary Material S1

Study Questionnaire: Telemedicine Implementation in Albania

Original Albanian wording with English translation

The questionnaire was administered online in Albanian. This supplementary document reproduces the original item wording from the study dataset and provides an English translation for publication transparency. Likert items used a five-point agreement response format from “Aspak dakord” (strongly disagree) to “Plotësisht dakord” (strongly agree).

Section A. Sociodemographic and Professional Characteristics

No.	Original Albanian item	English translation
1	Timestamp	Timestamp
2	Profesioni juaj:	Your profession
3	Qarku në të cilin punoni:	Region in which you work
4	Mosha juaj në vite	Your age in years
5	Niveli arsimor	Educational level
6	Vitet e përvojës profesionale	Years of professional experience
7	Niveli i kujdesit shëndetësor ku ushtroni profesionin tuaj	Level of healthcare in which you practice
8	A keni përvojë të mëparshme me përdorimin e telemjekësisë?	Do you have previous experience with the use of telemedicine?

Section B. Telemedicine Implementation Items

Response scale: 1 = Aspak dakord (Strongly disagree); 2 = Pjesërisht dakord; 3 = Neutral; 4 = Dakord (Agree); 5 = Plotësisht dakord (Strongly agree).

No.	Original Albanian item	English translation
1	Infrastruktura teknologjike në institucionin tim është e pamjaftueshme për zbatimin e telemjekësisë.	Technological infrastructure in my institution is insufficient for telemedicine implementation
2	Pajisjet teknologjike (kompjuterë, kamera, lidhje interneti) nuk janë të përshtatshme për shërbime telemjekësore.	Technological devices (computers, cameras, internet connection) are not adequate for telemedicine services
3	Problemet teknike pengojnë përdorimin e rregullt të telemjekësisë	Technical problems hinder regular telemedicine use
4	Sistemet ekzistuese informatike nuk janë të integruara me platformat e telemjekësisë.	Existing information systems are not integrated with telemedicine platforms
5	Siguria dhe mbrojtja e të dhënave të pacientëve përbën një shqetësim të rëndësishëm.	Data security and protection of patient information are major concerns
6	Nuk kam marrë trajnim të mjaftueshëm për përdorimin e telemjekësisë	I have not received sufficient training for telemedicine use
7	Personeli shëndetësor ka nevojë për trajnime të vazhdueshme mbi telemjekësinë.	Healthcare staff need continuous telemedicine training
8	Mungesa e aftësive digjitale përbën pengesë për zbatimin e telemjekësisë.	Lack of digital skills is a barrier to telemedicine implementation
9	Telemjekësia kërkon ndryshime në mënyrën tradicionale të ofrimit të kujdesit shëndetësor.	Telemedicine requires changes in traditional healthcare delivery
10	Ndihem i/e sigurt profesionalisht në përdorimin e telemjekësisë në praktikën time klinike	I feel professionally confident using telemedicine in clinical practice
11	Telemjekësia rrit ngarkesën e punës së personelit shëndetësor.	Telemedicine increases healthcare staff workload
12	Integrimi i telemjekësisë në rutinën e	Integrating telemedicine into daily work

	përditshme të punës është i vështirë.	routines is difficult
13	Telemjekësia kufizon marrëdhënien mjekâ€“pacient.	Telemedicine limits the healthcare professional-patient relationship
14	Jo të gjitha shërbimet shëndetësore janë të përshtatshme për telemjekësinë.	Not all healthcare services are suitable for telemedicine
15	Mungojnë protokolle dhe udhëzime të qarta për përdorimin e telemjekësisë.	Clear protocols and guidelines for telemedicine are lacking
16	Kuadri ligjor aktual nuk e mbështet mjaftueshëm zbatimin e telemjekësisë.	The current legal framework does not sufficiently support telemedicine implementation
17	Mungojnë politika të qarta shëndetësore për integrimin e telemjekësisë.	Clear health policies for telemedicine integration are lacking
18	Rimbursimi financiar i shërbimeve telemjekësore është i paqartë ose i pamjaftueshëm.	Financial reimbursement for telemedicine services is unclear or insufficient
19	Përgjegjësia ligjore në shërbimet telemjekësore nuk është e qartë.	Legal responsibility in telemedicine services is unclear
20	Mbështetja institucionale dhe menaxheriale për telemjekësinë është e pamjaftueshme.	Institutional and managerial support for telemedicine is insufficient
21	Telemjekësia përmirëson aksesin e pacientëve në shërbimet shëndetësore.	Telemedicine improves patient access to healthcare services
22	Telemjekësia ndihmon në përdorimin më efikas të kohës dhe burimeve.	Telemedicine supports more efficient use of time and resources
23	Telemjekësia mund të përmirësojë cilësinë e kujdesit shëndetësor.	Telemedicine may improve quality of healthcare
24	Telemjekësia është veÃ§anërisht e dobishme për zonat rurale ose të largëta.	Telemedicine is particularly useful for rural or remote areas
25	Jam i/e gatshëm/me ta përdor telemjekësinë në praktikën time të ardhshme.	I am willing to use telemedicine in my future practice

Section C. Open-Ended Questions

No.	Original Albanian item	English translation
1	Cilat mendoni se janë barrierat kryesore për integrimin e telemjekësisë në institucionin tuaj?	What do you think are the main barriers to integrating telemedicine in your institution?
2	Ã‡farë masash ose ndërhyrjesh do të ndihmonin më shumë në zbatimin efektiv të telemjekësisë?	What measures or interventions would help most with effective telemedicine implementation?
